# Gitelman syndrome with primary hyperparathyroidism: A case report

**DOI:** 10.1097/MD.0000000000039447

**Published:** 2024-08-23

**Authors:** Shanshen Yu, Jia Sun, Lijun Mou

**Affiliations:** a Linping Campus, The Second Affiliated Hospital of Zhejiang University School of Medicine, Hangzhou, China; b Division of Nephrology, The Second Affiliated Hospital of Zhejiang University School of Medicine, Hangzhou, China.

**Keywords:** calcium pyrophosphate deposition, Gitelman syndrome, hypercalcemia, primary hyperparathyroidism.

## Abstract

**Background:**

Gitelman syndrome (GS) is a rare autosomal recessive inherited salt-losing tubulopathy, typically devoid of hypercalcemia. Herein, we described one patient of GS presenting with hypercalcemia concomitant with primary hyperparathyroidism (PHPT).

**Methods:**

On September 28, 2020, a middle-aged female patient was admitted to our hospital with a 12-year history of hypokalemia and hypomagnesemia. Laboratory examinations unveiled hypokalemia with renal potassium wasting, hypomagnesemia, metabolic alkalosis, hypocalciuria, and gene sequencing revealed a homozygous mutation in *SLC12A3* (c.179C > T [p.T60M]). Subsequently, the diagnosis of GS was confirmed. In addition, the patient exhibited hypercalcemia and elevated levels of parathyroid hormone. Parathyroid ultrasound revealed left parathyroid hyperplasia, consistent with PHPT. Following aggressive treatment with potassium chloride and magnesium oxide, her serum potassium rose to 3.23 mmol/L, serum magnesium was 0.29 mmol/L, and her joint pain was relieved.

**Results:**

Based on the patient’s medical history, laboratory findings, and gene sequencing results, the definitive diagnosis was GS concomitant with PHPT.

**Conclusion:**

PHPT should be taken into consideration when patients diagnosed with GS exhibit hypercalcemia. While the serum potassium level readily exceeded the target threshold, correcting hypomagnesemia proved challenging, primarily because PHPT augments urinary magnesium excretion.

## 1. Introduction

Gitelman syndrome (GS) is a rare autosomal recessive inherited salt-losing tubulopathy because of the inactivating mutations in the *SLC12A3* gene, which encodes the sodium-chloride cotransporter of distal convoluted tubules. GS is characterized by chronic hypokalemia, metabolic alkalosis, hypomagnesemia, and hypocalciuria.^[1,2]^ Patients with GS typically do not present with hypercalcemia due to hypomagnesemia-induced parathyroid hormone (PTH) secretion dysfunction and end-organ resistance.^[3–7]^ Primary hyperparathyroidism (PHPT) is typically characterized by hypercalcemia associated with inappropriately elevated PTH levels.^[8]^ Therefore, in instances where hypercalcemia emerges in GS, consideration of PHPT is warranted. Herein, we described one patient of GS presenting with hypercalcemia concomitant with PHPT.

## 2. Case pressentation

On September 28, 2020, a 46-year-old female patient was admitted to our hospital presenting with a 12-year history of hypokalemia and hypomagnesemia. She has manifested symptoms including salt cravings, polydipsia, nocturia, paroxysmal fatigue, and numbness in her hands since childhood. Twelve years ago, her prenatal care revealed hypokalemia with the lowest recorded serum potassium concentration being 2.2 mmol/L. Intermittent swelling and pain in both ankles commenced 2 years ago, and intermittent neck and left shoulder pain occurred 1 year ago. On August 18, 2020, the patient was hospitalized in a local hospital, with her blood pressure measured at 123/66 mm Hg postadmission. Laboratory tests revealed hypokalemia due to renal potassium wasting, hypomagnesemia, metabolic alkalosis, hypocalciuria, hypercalcemia, elevated PTH level, hyperreninemia (Table 1), and normal bone mineral density assessed via dual-energy X-ray absorptiometry of the hip joint and lumbar spine. Consequently, she was admitted to our hospital.

**Table 1:** Laboratory tests.

Examination item	08.18	09.28	10.26	12.14	Reference value
Serum biochemistry					
Potassium, mmol/L	2.74	3.07	3.15	3.23	3.50–5.50
Magnesium, mmol/L	0.34	0.29	0.35	0.29	0.77–1.77
Calcium, mmol/L	2.90	2.94	2.91	2.99	2.20–2.65
Sodium, mmol/L		140.8	137.2	136.8	135.0–145.0
Chloride, mmol/L	97.4	99.0	98.4	97.8	96.0–106.0
Phosphate, mmol/L		1.10	1.04	0.8	0.81–1.45
PTH, pg/mL	122.0	105.0	107.9	102.2	15.0–65.0
Arterial blood gas analysis					
PH	7.451	7.479			7.35–7.45
PaCO2	37.7	37.1			36.0–44.0
HCO3−	25.7	27.3			22.0–26.0
Potassium, mmol/L	2.86	2.8			3.5.0–5.5.0
Ionized calcium, mmol/L	1.37	1.39			1.15–1.29
Sodium, mmol/L		141.0			135.0–145.0
Chloride, mmol/L		102.0			96.0–106.0
AG, mmol/L	16.5	11.0			8.0–16.0
Urinary potassium/creatinine, mmol/mmol		6.034			
Urinary sodium, mmol		152.7			
Urinary chloride, mmol		133.3			
Urinary calcium/creatinine, mmol/mmol		0.139			
24-h urine tests					
Potassium, mmol/24 h	94.7				25.00–100.0
Sodium, mmol/24 h	78.4				130.0–260.0
Calcium, mmol/24 h	1.24				2.50–7.50
Chloride, mmol/24 h	127.8				100.0–250.0
Plasma renin, pg/mL	38.93				4.00–24.00
Plasma aldosterone, pg/mL	266.27				10.0–160.0
Angiotensin II, pg/mL	172.56				25.0–129.0

AG = anion gap, HCO3− = bicarbonate ions, PaCO2 = partial pressure of carbon dioxide in arterial blood, PH = potential of hydrogen, PTH = parathyroid hormone.

She had a history of type 2 diabetes for more than 10 years and was prescribed sitagliptin and glimepiride for glycemic control. Her parents were healthy and nonconsanguineous marriage.

**Figure 1.**
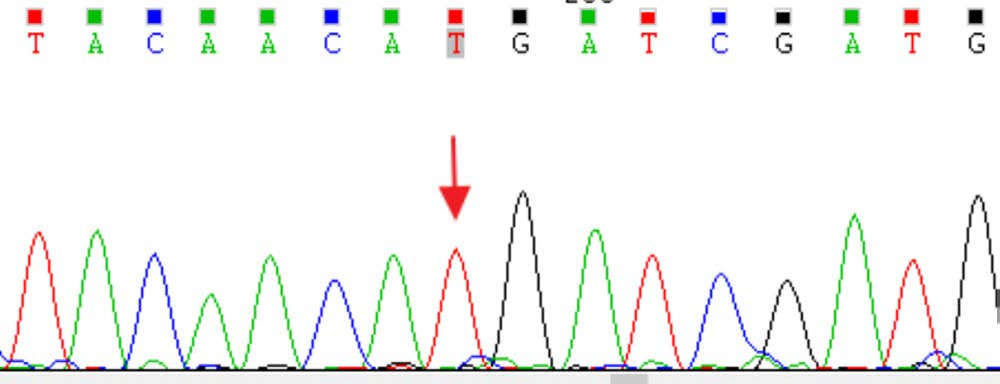
Gene analysis results.

Therefore, a next-generation sequencing-based panel was performed to identify the exact type of salt-losing tubulopathies. The method of gene sequencing was performed as previously reported.^[9]^ A homozygous mutation in *SLC12A3* (c.179C > T [p. T60M]) was identified (Fig. 1). The p.T60M mutation is a hotspot in the Chinese GS cohort.^[10,11]^

To elucidate the etiology of hypercalcemia, further examinations were conducted: ultrasound of the parathyroid indicated left parathyroid hyperplasia; cervical spine computed tomography showed pyrophosphate deposition in the ligamentum flavum and calcification of the intervertebral disc annulus, while urinary tract ultrasound yielded normal findings.

Based on the patient’s medical history, laboratory findings, and gene sequencing results, the definitive diagnosis was GS concomitant with PHPT.

Potassium chloride tablets (4.5 g per day) and magnesium oxide (450 mg magnesium per day) were administered to correct hypokalemia and hypomagnesemia. 2017 Kidney Disease: Improving Global Results guidelines suggest that the serum potassium and magnesium levels of patients with GS should be maintained at a minimum of 3.0 and 0.6 mmol/L, respectively.^[2]^ On October 26, 2020, the patient’s joint pain was relieved, and her serum magnesium level rose to 0.35 mmol/L; however, hypomagnesemia persisted (Table 1). Therefore, the dose of magnesium was increased to 600 mg per day. However, achieving the target serum magnesium concentration remained challenging for the patient until December 14, 2020.

## 3. Discussion and conclusion

The patient, a middle-aged woman, had been afflicted with salt cravings, polydipsia, and paroxysmal fatigue since childhood. Laboratory tests showed hypokalemia due to renal potassium wasting, hypomagnesemia, metabolic alkalosis, and hypocalciuria. Gene sequencing identified a homozygous mutation in *SLC12A3*, indicating the presence of GS. In addition, she exhibited hypercalcemia, elevated PTH, and parathyroid hyperplasia, and PHPT was identified. Following magnesium supplementation, her joint pain subsided.

Hypomagnesemia constitutes a key clinical criterion for the diagnosis of GS. Generally, patients with GS do not present hypercalcemia, with impaired parathyroid gland function being proposed as a major factor associated with magnesium deficiency. In 1973, a study provided support for the hypothesis that magnesium depletion causes impaired synthesis or secretion of PTH.^[6]^ In 1995, Bianchetti et al^[3]^ confirmed the renal origin of magnesium depletion and disclosed that the relationship between PTH and ionized calcium concentrations was blunted in patients with GS, thus providing the first evidence that patients with GS have a disturbed secretion of PTH, which seems to be due to chronic hypomagnesemia in GS. In addition, this relationship was substantiated by the observation of a marked elevation in serum PTH level and the subsequent normalization of calcium levels in a patient with GS after intravenous infusion of magnesium salts. The steep rise in serum PTH induced by intravenous magnesium (detectable within 1 minute) indicated that PTH secretion, rather than synthesis, was impaired by magnesium deficiency.^[4]^ In addition to the impaired release of PTH, Rude et al^[5]^ noted that during the course of magnesium repletion, the serum calcium concentration failed to rise significantly within the initial 24 hours despite high-normal to greatly elevated serum PTH concentrations. The delayed response to endogenous PTH implies the existence of skeletal end-organ resistance to PTH.^[5]^ Therefore, this patient with GS presented with profound hypomagnesemia, accompanied by hypercalcemia and elevated PTH levels, suggestive of PHPT.

In addition to elevated serum calcium levels, hypomagnesemia stands as a common electrolyte disturbance among patients with PHPT. Hypomagnesemia is present in approximately 25.1% of patients with PHPT.^[12]^ Urinary magnesium excretion tends to be slightly increased in patients with PHPT, which may be associated with a reversible defect in renal magnesium conservation.^[13–15]^

The patient’s cervical spine computed tomography showed pyrophosphate deposition in the ligamentum flavum and calcification of the intervertebral disc annulus. Calcium pyrophosphate deposition (CPPD) is a common crystalline joint disease caused by calcium pyrophosphate crystal deposition in joints and adjacent tissues.^[16–18]^ Chondrocalcinosis, defined as calcification of the cartilage and identified by radiological or histological examinations, is a common feature of CPPD.^[17,19,20]^ Several documented cases have reported CPPD within the ligamentum flavum,^[21,22]^ as well as deposition in lumbar disc fibrocartilage.^[23]^

CPPD was identified as a manifestation of GS.^[24]^ In GS, low serum magnesium results in calcium pyrophosphate formation in soft tissues and joints by inhibition of pyrophosphate hydrolysis and reducing crystal solubility.^[25]^ A cohort of 57 patients with GS revealed a significant association between GS and extensive chondrocalcinosis, with the highest prevalence of cervical vertebra, particularly the C1-C2 level.^[26]^

CPPD can also be induced by hyperparathyroidism. A prospective study conducted in 1978 revealed the relationship between this endocrinopathy and calcium pyrophosphate dihydrate crystal deposition disease among patients with PHPT.^[27]^ In a Japanese cohort of 132 patients with PHPT, chondrocalcinosis was identified in 6.1% of cases. These patients exhibited higher serum calcium levels and were older than those without this disorder, corroborating an earlier finding,^[28]^ suggesting that chondrocalcinosis is caused by the cumulative impact of persistent hypercalcemia and age-related changes in articular cartilage.^[29]^ A study conducted in the United States involving 25,157 patients diagnosed with CPPD, with an average age of 68.1 ± 12.3 years, demonstrated that hyperparathyroidism exhibited the most significant positive correlation with CPPD.^[30]^ In 2011, the European League Against Rheumatism published a meta-analysis confirming a strong association between hyperparathyroidism and CPPD.^[31]^

In summary, hypomagnesemia was a concomitant consequence of both GS and PHPT, making it challenging for the patient to achieve standard serum magnesium levels. CPPD was caused by the combined effects of PHPT and hypomagnesemia. Previously, we proposed parathyroid surgery for the patient. Meanwhile, we reinforced aggressive magnesium supplements to mitigate the deposition of calcium pyrophosphate resulting from hypomagnesemia. Subsequent to treatment, the patient experienced relief from symptoms and declined surgical intervention. This constitutes a limitation of this case report.

Despite hypocalciuria, hypercalcemia is generally absent in patients with GS due to magnesium deficiency. The presence of hypercalcemia in patients with GS warrants consideration of PHPT. Furthermore, PHPT can elevate urinary magnesium excretion, complicating the correction of hypomagnesemia in patients with GS. Considering the potential risk of worsening PHPT and hypercalcemia after the correction of hypomagnesemia, the management of GS requires individualized approaches.

## Author contributions

**Formal analysis:** Shanshen Yu.

**Investigation:** Shanshen Yu.

**Visualization:** Shanshen Yu.

**Writing – original draft:** Shanshen Yu.

**Writing – review & editing:** Shanshen Yu, Lijun Mou.

**Conceptualization:** Lijun Mou.

**Methodology:** Lijun Mou.

**Validation:** Lijun Mou.

**Data curation:** Jia Sun.

**Software:** Jia Sun.

**Formal analysis:** Shanshen Yu.

**Investigation:** Shanshen Yu.

**Visualization:** Shanshen Yu.

**Writing – original draft:** Shanshen Yu.

**Writing – review & editing:** Shanshen Yu, Lijun Mou.

**Conceptualization:** Lijun Mou.

**Methodology:** Lijun Mou.

**Validation:** Lijun Mou.

**Data curation:** Jia Sun.

**Software:** Jia Sun.

## References

[1] Filippatos T Rizos C Tzavella E Elisaf M. Gitelman syndrome: an analysis of the underlying pathophysiologic mechanisms of acid-base and electrolyte abnormalities. Int Urol Nephrol. 2018;50:91–6. 28744758 10.1007/s11255-017-1653-4

[2] Blanchard A Bockenhauer D Bolignano D. Gitelman syndrome: consensus and guidance from a Kidney Disease: Improving Global Outcomes (KDIGO) Controversies Conference. Kidney Int. 2017;91:24–33. 28003083 10.1016/j.kint.2016.09.046

[3] Bianchetti M Bettinelli A Casez J. Evidence for disturbed regulation of calciotropic hormone metabolism in Gitelman syndrome. J Clin Endocrinol Metab. 1995;80:224–8. 7829616 10.1210/jcem.80.1.7829616

[4] Pantanetti P Arnaldi G Balercia G Mantero F Giacchetti G. Severe hypomagnesaemia-induced hypocalcaemia in a patient with Gitelman’s syndrome. Clin Endocrinol (Oxf). 2002;56:413–8. 11940055 10.1046/j.1365-2265.2002.01223.x

[5] Rude R Oldham S Singer F. Functional hypoparathyroidism and parathyroid hormone end-organ resistance in human magnesium deficiency. Clin Endocrinol (Oxf). 1976;5:209–24. 182417 10.1111/j.1365-2265.1976.tb01947.x

[6] Suh S Tashjian A Matsuo N Parkinson D Fraser D. Pathogenesis of hypocalcemia in primary hypomagnesemia: normal end-organ responsiveness to parathyroid hormone, impaired parathyroid gland function. J Clin Invest. 1973;52:153–60. 4345201 10.1172/JCI107159 PMC302237

[7] Fatemi S Ryzen E Flores J Endres D Rude R. Effect of experimental human magnesium depletion on parathyroid hormone secretion and 1,25-dihydroxyvitamin D metabolism. J Clin Endocrinol Metab. 1991;73:1067–72. 1939521 10.1210/jcem-73-5-1067

[8] Applewhite M Schneider D. Mild primary hyperparathyroidism: a literature review. Oncologist. 2014;19:919–29. 25063228 10.1634/theoncologist.2014-0084 PMC4153463

[9] Mou L Wu F. Simultaneous Homozygous Mutations in *SLC12A3* and *CLCNKB* in an Inbred Chinese Pedigree. Genes. 2021;12:369. 33807568 10.3390/genes12030369 PMC7999423

[10] Shen Q Chen J Yu M. for Chinese Children Genetic Kidney Disease Database (CCGKDD). Multi-centre study of the clinical features and gene variant spectrum of Gitelman syndrome in Chinese children. Clin Genet. 2021;99:558–64. 33382082 10.1111/cge.13913

[11] Zhong F Ying H Jia W. Characteristics and follow-up of 13 pedigrees with Gitelman syndrome. J Endocrinol Invest. 2019;42:653–65. 30413979 10.1007/s40618-018-0966-1 PMC6531408

[12] Na D Tao G Shu-Ying L. Association between hypomagnesemia and severity of primary hyperparathyroidism: a retrospective study. BMC Endocr Disord. 2021;21:170. 34416890 10.1186/s12902-021-00838-y PMC8379767

[13] Machado N Wilhelm S. Diagnosis and evaluation of primary hyperparathyroidism. Surg Clin North Am. 2019;99:649–66. 31255197 10.1016/j.suc.2019.04.006

[14] Sutton RAL. Plasma magnesium concentration in primary hyperparathyroidism. Br Med J. 1970;1:529–33. 5436362 10.1136/bmj.1.5695.529 PMC1699502

[15] Hulter H Peterson J. Renal and systemic magnesium metabolism during chronic continuous PTH infusion in normal subjects. Metabolism. 1984;33:662–6. 6738367 10.1016/0026-0495(84)90067-2

[16] Iqbal SM Qadir S Aslam HM Qadir MA. Updated treatment for calcium pyrophosphate deposition disease: an insight. Cureus. 2019;11:e3840. 30891381 10.7759/cureus.3840 PMC6411330

[17] Bencardino J Hassankhani A. Calcium pyrophosphate dihydrate crystal deposition disease. Semin Musculoskelet Radiol. 2003;7:175–85. 14593559 10.1055/s-2003-43228

[18] Rosales-Alexander JL Balsalobre Aznar J Magro-Checa C. Calcium pyrophosphate crystal deposition disease: diagnosis and treatment. Open Access Rheumatol. 2014;6:39–47. 27790033 10.2147/OARRR.S39039 PMC5045115

[19] Williams C Rosenthal A. Pathogenesis of calcium pyrophosphate deposition disease. Best Pract Res Clin Rheumatol. 2021;35:101718. 34696986 10.1016/j.berh.2021.101718

[20] Joshi A Siva C. Magnesium disorders can cause calcium pyrophosphate deposition disease: a case report and literature review. Eur J Rheumatol. 2018;5:53–7. 29657876 10.5152/eurjrheum.2017.16116 PMC5895153

[21] Brown T Quinn S D’Agostino A. Deposition of calcium pyrophosphate dihydrate crystals in the ligamentum flavum: evaluation with MR imaging and CT. Radiology. 1991;178:871–3. 1994435 10.1148/radiology.178.3.1994435

[22] Delamarter R Sherman J Carr J. Lumbar spinal stenosis secondary to calcium pyrophosphate crystal deposition (pseudogout). Clin Orthop Relat Res. 1993;289:127–30. 8472402

[23] Ellman M Vazques L Brown N Mandel N. Calcium pyrophosphate dihydrate deposition in lumbar disc fibrocartilage. J Rheumatol. 1981;8:955–8. 6276538

[24] Abhishek A Doherty M. Epidemiology of calcium pyrophosphate crystal arthritis and basic calcium phosphate crystal arthropathy. Rheum Dis Clin North Am. 2014;40:177–91. 24703342 10.1016/j.rdc.2014.01.002

[25] Ham Y Mack H Colville D Harraka P Savige J. Gitelman syndrome and ectopic calcification in the retina and joints. Clin Kidney J. 2021;14:2023–8. 34476088 10.1093/ckj/sfab034 PMC8406063

[26] Chotard E Blanchard A Ostertag A. Calcium pyrophosphate crystal deposition in a cohort of 57 patients with Gitelman syndrome. Rheumatology. 2021;61:2494–503. 10.1093/rheumatology/keab578 34508565

[27] Rynes R Merzig E. Calcium pyrophosphate crystal deposition disease and hyperparathyroidism: a controlled, prospective study. J Rheumatol. 1978;5:460–8. 216803

[28] McGill PE Grange AT Royston CS. Chondrocalcinosis in primary hyperparathyroidism. Influence of parathyroid activity and age. Scand J Rheumatol. 1984;13:56–8. 6719062 10.3109/03009748409102668

[29] Yashiro T Okamoto T Tanaka R. Prevalence of chondrocalcinosis in patients with primary hyperparathyroidism in Japan. Endocrinol Jpn. 1991;38:457–64. 1843264 10.1507/endocrj1954.38.457

[30] Kleiber Balderrama C Rosenthal A Lans D Singh J Bartels C. Calcium pyrophosphate deposition disease and associated medical comorbidities: a national cross-sectional study of US Veterans. Arthritis Care Res (Hoboken). 2017;69:1400–6. 27898996 10.1002/acr.23160 PMC5472491

[31] Zhang W Doherty M Bardin T. European League Against Rheumatism recommendations for calcium pyrophosphate deposition. Part I: terminology and diagnosis. Ann Rheum Dis. 2011;70:563–70. 21216817 10.1136/ard.2010.139105

